# Gene discovery using massively parallel pyrosequencing to develop ESTs for the flesh fly *Sarcophaga crassipalpis*

**DOI:** 10.1186/1471-2164-10-234

**Published:** 2009-05-19

**Authors:** Daniel A Hahn, Gregory J Ragland, D DeWayne Shoemaker, David L Denlinger

**Affiliations:** 1 Department of Entomology and Nematology, The University of Florida, PO Box 110620, Gainesville, Fl, 32611-0620, USA; 2 USDA ARS, Center for Medical, Agricultural, and Veterinary Entomology, 1600 SW 23rd Drive, Gainesville, FL 32608, USA; 3 Department of Entomology, Ohio State University, 400 Aronoff Laboratory, 318 West 12th Avenue, Columbus, OH 43210, USA

## Abstract

**Background:**

Flesh flies in the genus *Sarcophaga *are important models for investigating endocrinology, diapause, cold hardiness, reproduction, and immunity. Despite the prominence of *Sarcophaga *flesh flies as models for insect physiology and biochemistry, and in forensic studies, little genomic or transcriptomic data are available for members of this genus. We used massively parallel pyrosequencing on the Roche 454-FLX platform to produce a substantial EST dataset for the flesh fly *Sarcophaga crassipalpis*. To maximize sequence diversity, we pooled RNA extracted from whole bodies of all life stages and normalized the cDNA pool after reverse transcription.

**Results:**

We obtained 207,110 ESTs with an average read length of 241 bp. These reads assembled into 20,995 contigs and 31,056 singletons. Using BLAST searches of the NR and NT databases we were able to identify 11,757 unique gene elements (E<0.0001) representing approximately 9,000 independent transcripts. Comparison of the distribution of *S. crassipalpis *unigenes among GO Biological Process functional groups with that of the *Drosophila melanogaster *transcriptome suggests that our ESTs are broadly representative of the flesh fly transcriptome. Insertion and deletion errors in 454 sequencing present a serious hurdle to comparative transcriptome analysis. Aided by a new approach to correcting for these errors, we performed a comparative analysis of genetic divergence across GO categories among *S. crassipalpis*, *D. melanogaster*, and *Anopheles gambiae*. The results suggest that non-synonymous substitutions occur at similar rates across categories, although genes related to response to stimuli may evolve slightly faster. In addition, we identified over 500 potential microsatellite loci and more than 12,000 SNPs among our ESTs.

**Conclusion:**

Our data provides the first large-scale EST-project for flesh flies, a much-needed resource for exploring this model species. In addition, we identified a large number of potential microsatellite and SNP markers that could be used in population and systematic studies of *S. crassipalpis *and other flesh flies.

## Background

Flesh flies in the genus *Sarcophaga *(Diptera: Sarcophagidae) have long been important models in insect physiology and biochemistry, particularly with respect to the study of endocrinology [1,2], diapause [3,4], cold hardiness [5,6], reproduction [7,8], and immunity [9]. Larvae of *Sarcophaga *flies typically feed on carrion or feces, playing an important ecological role in decomposition and feature prominently in human forensic studies [10,11]. *Sarcophaga *species can cause myiasis in humans and livestock and therefore are of importance to medical and veterinary entomology [12]. In addition, *Sarcophaga *flies are natural hosts of the parasitic wasp *Nasonia vitripennis *and are often used in laboratory studies and for wasp culture. *Nasonia vitripennis *parasitizes pupae of many species of higher flies, including house flies and other filth flies, making them economically important for biological control [13]. Furthermore, *N. vitripennis *is a popular model for understanding host-parasite relationships, behavioral ecology, development, and evolution, thus offering an impetus for sequencing the genome of this wasp and two congeners *N. giraulti *and *N. longicornis *[14-16].

Despite its prominence as a model in several sub-disciplines, there is currently no genome project for any *Sarcophaga *species, and genomic resources are limited. Several laboratories have performed low-throughput EST projects such as differential display [17] and subtractive hybridization [18], but the mainstay of functional genomic research on species in this genus has focused on homology-based, gene-by-gene cloning. The genomic resources developed for a handful of insects including *Drosophila*, honey bees, mosquitoes, and *Bombyx mori *has prompted much work on these species, pushing our understanding of processes in these few models far ahead of other insects. Development of such resources for *Sarcophaga *would greatly benefit the study of flesh flies as has been done with other non-traditional model insects such as *Polistes *wasps [19], Glanville fritillary butterflies [20], and tobacco hornworm moths [21].

We describe an EST collection developed as a genomic resource for the flesh fly *Sarcophaga crassipalpis*, a model species for studying numerous processes including diapause, reproduction, endocrinology, stress tolerance, and immunity. To facilitate identifying sets of genes involved in a broad range of processes we developed our EST set from a normalized whole-body library of numerous *S. crassipalpis *life stages including diapause-destined and non diapause-destined larvae, diapausing and non-diapausing pupae, and reproductive and non-reproductive adults of both sexes (Table 1). We confidently identified approximately 9,000 different transcripts involved in numerous biological processes. Within these transcripts, we identified more than 500 potential microsatellite loci and over 12,000 SNPs that can be used for future population studies. Comparison of the distribution of *S. crassipalpis *unigenes among GO Biological Process functional groups with that of the *Drosophila melanogaster *transcriptome suggests that our ESTs are broadly representative of the flesh fly transcriptome. Combining our data with full transcriptomes from *D. melanogaster *and *A. gambiae*, we used a three-species comparison to test whether coding sequences are evolving at similar rates across these GO categories.

**Table 1:** RNA Extraction Strategy.

Pre-adult stages		Adult Stages	
Exposed to short days to induce diapause development	Exposed to long days to induce direct development	Non-reproductive Fed only sugar	Reproductive Fed sugar and protein (liver)
First Instar Larvae	First Instar Larvae	Day 1 Males	Day 1 Males
Second Instar Larvae	Second Instar Larvae	Day 1 Females	Day 1 Females
Early Third Instar Larvae	Early Third Instar Larvae	Day 2 Males	Day 2 Males
Late Third Instar Larvae	Late Third Instar Larvae	Day 2 Females	Day 2 Females
Wandering Larvae	Wandering Larvae	Day 3 Males	Day 3 Males
Pupae	Early Diapause Pupae (15 days)	Day 3 Females	Day 3 Females
Early Pharate Adult	Mid-diapause Pupae (30 days)	Day 4 Males	Day 4 Males
Mid-Pharate Adult (red eye stage)	Late Diapause Pupae (60 Days)	Day 4 Females	Day 4 Females
Late Pharate Adult (black bristle stage)		Day 5 Males	Day 5 Males
		Day 5 Females	Day 5 Females
		Day 7 Males	Day 7 Males
		Day 7 Females	Day 7 Females
		Day 9 Males	Day 9 Males
		Day 9 Females	Day 9 Females

Flesh fly life stages that were individually RNA extracted and combined equally prior to cDNA library synthesis and normalization.

## Results and discussion

Our normalized *S. crassipaplis *library was first subjected to a one-quarter plate titration run on the 454 GS-FLX machine that yielded 1,512,233 total bases from 5,727 sequence reads that assembled into 554 contigs and 3,182 singletons. Of these contigs and singletons 1,465 were identifiable at an E ≤ 0.001 using BLASTX against the NCBI non-redundant protein database (NR).

We followed this one-quarter plate preliminary run with a full-plate production run that yielded 72,816,811 total bases from 281,537 sequence reads and combined the results of both runs into a single dataset which was assembled *de novo*. Files containing our results are available from the National Center for Biotechnology Information Short Read Archive (accession numbers SRR005065 and SRR006884). After filtering adaptors and low-quality sequences [22] we were left with 74,329,044 total bases from 207,110 sequence reads with an average length of 241 bases. These data were assembled into 20,995 contigs with a mean length of 332 bp and a range of 30 to 2,958 bp as well as an additional 31,056 singletons for a total of 52,051 high-quality sequences. A file of all assembled contigs and singletons is available from the authors upon request.

Subjecting these sequences to a BLASTX search against the NCBI-NR protein database yielded 19,609 well-identified sequences with at least one hit E ≤ 0.0001 (37%), 15,241 poorly-identified sequences with hits between E = 0.0001 and E = 10, and 32,433 with no useful hits (E>10). Subjecting these sequences to a BLASTN search against the NCBI-NT sequence database yielded 6,321 well-identified sequences with a hit E ≤ 0.0001 (12%), 44,268 poorly-identified sequences with hits between E = 0.0001 and E = 10, and 1,453 with no useful hits (E>10). Well-identified sequences (E<0.001) from both searches were combined and compared with each other using BLASTN, reducing the number of well-identified sequences into 11,757 unique gene elements or unigenes. To estimate how many of these unigenes represented unique transcripts as opposed to different non-contiguous fragments of the same transcript we produced a file of the top 50 hits for each unigene and used these lists to compare accession numbers that overlapped among different unigenes with an E<0.001 using a custom Excel macro followed by manual editing. This procedure identified 9,317 sequences that are likely independent transcripts (Table 2).

**Table 2:** Summary of *Sarcophaga crassipalpis *EST data.

Total Bases	74,329,044
High-quality Reads	207,110
Average Read Length	241
Number of Contigs	20,995
Average Contig Length	332
Range Contig Length	30 to 2,958
Number of Reads in Contigs	180,974
Number of Singletons	31,056
Unigenes with E ≤ 0.001 vs. NR or NT Database	11,757
Estimated Unique Transcripts	9,317

While the ~9,000 identified transcripts are certainly less than the entire transcriptome, we acquired many sequences of interest. Because our groups, among others, are particularly interested in diapause and stress biology, we compiled a brief list of genes we expect play a role in diapause (Additional file 1). Clearly, even this abbreviated list will provide fodder for years of functional genomics work on these candidate genes for our group and others.

**Figure 1:**
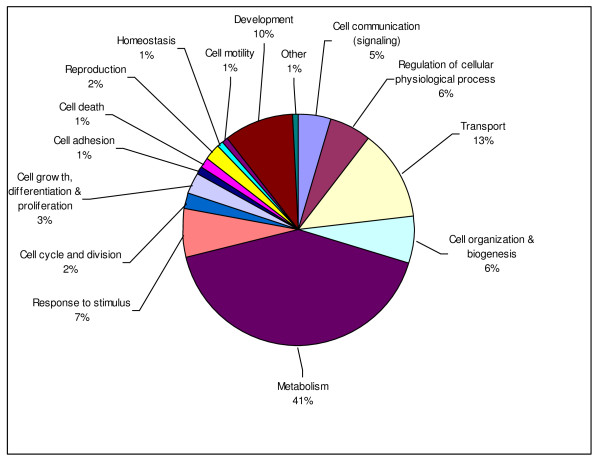
***Sarcophaga crassipalpis *sequences were classified into one of 14 major sub-categories within the Biological Processes GO category**.

**Figure 2:**
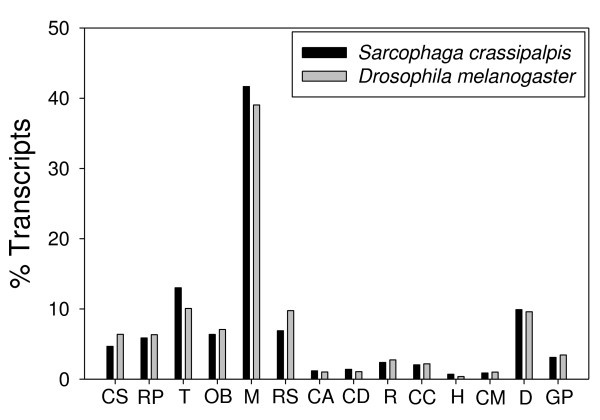
**A comparison of the distribution across 14 major Biological Process GO sub-classes in our *Sarcophaga crassipalpis *library versus predicted ESTs from *Drosophila melanogaster***. The sub-categories are CS = cell communication (signaling), RP = Regulation of cellular physiological process, T = Transport, OB = Cell organization and biogenesis, M = Metabolism, RS = Response to stimulus, CA = Cell adhesion, CD = Cell death, R = Reproduction, CC = Cell cycle and division, H = Homeostasis, CM = Cell motility, D = Development, and GP = Cell growth, differentiation, and proliferation.

Our major goal was to generate a substantial representative sample of the transcriptome. To assess whether our EST project was broadly representative of our expectations for the *Sarcophaga *transcriptome, we used the annotations for each of our contigs and singletons to assign it to one of 14 major GO categories for Biological Process, producing assignments for 9,468 out of a total 52,051 transcripts (Fig. 1). We also compared the distribution of our sequences among these 14 GO functional groups within Biological Process to the distribution of the 15,183 predicted genes for *Drosophila melanogaster*, 5,053 of which are placed into those same functional groups (Fig. 2, FlyBase, version 2008_07). Although there are noticeable differences between the *Drosophila *and *Sarcophaga *data in the percentage of transcripts within a few sub-categories, concordance in the overall distributions suggests that our library sampled widely across sub-categories and provides a good representation of the *S. crassipalpis *transcriptome. As sequencing technologies improve, further exploration of the *S. crassipalpis *transcriptome will likely include sequencing of new libraries made from key tissues during focal life stages that may not have high representation in total body extracts, such as brains from diapausing pupae or ovaries from pre and post-reproductive females, as well as deeper sequencing to both reveal rare transcripts and provide the additional information necessary to identify more of the unannotated transcript fragments in our current whole-body library.

**Figure 3:**
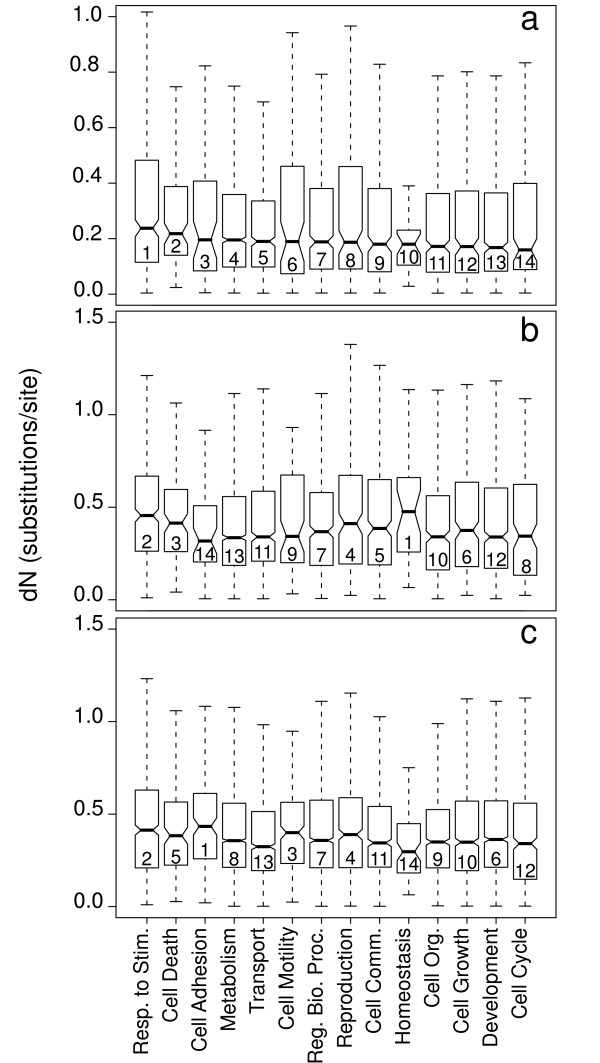
**Box plots of the distributions of non-synonymous substitution rate (dN) values in each GO category**. The pairwise comparisons are: *S. crassipalpis *to *D. melanogaster *(a), *S. crassipalpis *to *A. gambiae *(b), and *A. gambiae *to *D. melanogaster *(c). The upper and lower bounds of the notches represent the 95% confidence interval of the median, and the numbers inside the boxes represent the relative rank within each pairwise comparison.

Because our EST database appears broadly representative of the *S. crassipalpis *transcriptome, we compared it to the available transcriptomes of *D. melanogaster *and *A. gambiae *to assess whether coding sequences were evolving at equivalent rates across GO categories. Non-synonymous rates of substitution (dN) were roughly gamma-distributed across genes within each GO category for all three pairwise comparisons. Median values across all GO categories were generally lowest in comparisons of *S. crassipalpis *to *D. melanogaster *(Fig. 3), reflecting their close relationship within Cyclorrapha relative to the relatively deep phylogenetic split between Cyclorrapha and Culicomorpha. The 95% confidence intervals of the median (notches in Fig. 3) did not differ substantially across categories within any of the three pairwise comparisons (similarly shaded bars in Fig. 3), suggesting largely homogeneous rates of amino acid substitution. However, the median dN for Response to stimuli had the highest (*D. melanogaster *to *A. gambiae*; *S. crassipalpis *to *A. gambiae*) or second highest (*S. crassipalpis *to *D. melanogaster*) value in the rank ordering among GO categories across all three pairwise comparisons. This consistently high dN across multiple comparisons of species diverged on the order of 200 million years ago [23] suggests that genes regulating responses to stimuli may evolve relatively rapidly across dipteran taxa. This divergence meshes well with obvious differences in sensory strategies between these three species that must identify and locate very different nutritional resources (rotting flesh, live animals for blood meals, and rotting fruit). In addition, responses to pheromonal and behavioural cues associated with reproduction vary widely across taxa and are likely under strong diversifying selection.

In addition to the focal animal, transcriptomics projects can also provide insights into parasites, pathogens, and other associated microorganisms. Because we used whole organisms grown in non-sterile culture conditions we expected a portion of our library to include microorganismal sequences, some which are directly associated with *S. crassipalpis *and some that are inadvertent environmental contaminants. We identified only 160 non-metazoan sequences that appeared to be independent transcripts with an E<0.0001 from our BLASTX and BLASTN searches. Although we expect that flesh flies have a rich microorgansimal community, our selection of poly-A RNA as the starting material for our library likely eliminated many potential microbial sequences. Few hits were ascribed to any single microorganismal taxa. Roughly one-third of the sequences had high sequence similarity to various eukaryotic microorganisms including yeasts (e.g., *Candida, Saccharomyces*), filamentous fungi (e.g., *Aspergillus, Neurospora*), and protozoans (e.g., *Plasmodium, Theileria*). Sequences having apparent bacterial origin included many genera with species that have been associated with insects either as pathogens or as normal flora in the gut (e.g., *Burkholderia *and *Escherichia*), but these sequences could just as easily represent environmental contamination. Perhaps most interesting of the non-metazoan sequences were those for transposable elements, particularly sequences in the *Mariner-Tc1 *family and several retroviral elements. With future development such sequences may provide insight into new functional genomics tools for *Sarcophaga*, potentially including transgenic modification.

Another interesting feature of our 454 sequence data is that a number of sequences appear to contain repeat motifs (i.e., microsatellites) or single nucleotide polymorphisms (SNPs). We searched for microsatellite motifs within our sequences using the program MSATCOMMANDER [24] to identify sequences with di-, tri-, tetra-, penta, and hexanucleotide repeats. Our search for repeat motifs revealed 521 sequences containing some such motif from the total set of 52,053 sequences (using a minimum repeat length of seven, Additional file 2). The high levels of variation typically found for microsatellite markers have led to their widespread use for a broad range of studies such as genome mapping, parentage analysis, and analyses of gene flow and genetic structure. Because our library was constructed from a long-standing laboratory colony expected to harbor little standing variation compared to field populations, we did not screen any of these loci for allelic variability. Similarly, to identify SNPs in our contigs we used the program PolyBayesShort, a version of the PolyBayes program [25] that has been optimized for data from short read platforms such as 454, under the default parameters. We found at total of 12,090 SNPs that were distributed among 3436 of our 20,995 contigs, yielding an average of 7.23 SNPs per 10 kb. However, because our samples were derived from a long-standing, inbred laboratory colony our SNP estimates are likely a small fraction of the variation existing in *S. crassipalpis *populations in the field. We hope that identification of candidate microsatellite and SNP loci will be useful for others interested in field-based population studies of *S. crassipalpis *and closely related *Sarcophaga *species, some of which are important for forensic studies.

## Conclusion

Studies of flesh flies in the genus *Sarcophaga *have contributed greatly to our understanding of diapause, stress resistance, reproduction, and immunity in insects, but work in this "non-traditional" model organism has been hampered by a lack of genomic resources. Our use of pyroseqencing to develop a new EST library containing approximately 9,000 transcripts provides a new resource for innovative work that we hope will draw renewed interest in using flesh flies as a model. We expect that these EST data will provide the foundation for a proliferation of functional work on flesh flies including "-omics" approaches such as the development of microarrays for large scale gene expression studies as well as serving as a foundation for identifying previously unrecognizable proteins in proteomics studies. These data will also increase our ability to explore specific gene and pathway-based approaches such as transcript quantification by qRT-PCR and RNA interference of candidate genes that have previously been refractory to homology-based cloning. Furthermore, these data compliment the growing number of EST and genome projects in flies other than *Drosophila *and mosquitoes, such as the medfly *Ceratitis capitata *and the Hessian fly *Mayetiola destructor *[26,27], enhancing resources for comparative studies of molecular evolution across the Diptera. Our initial comparative analysis of evolutionary rates across GO categories illustrates this utility, and we anticipate that similar genome and transcriptome-wide analyses will yield important insights into insect diversification.

## Methods

The culture of *S. crassipalpis *used for this work originated in Columbus, Ohio, and was maintained in the laboratory for nearly 8 years before the onset of this project. Non-diapausing pupae were obtained by rearing the colony under long-day conditions (15L:9D) with adults held at 25°C and larvae and pupae at 20°C, whereas diapausing pupae were generated by rearing the maternal generation of adults, larvae and pupae under short-day conditions (12L:12D) with adults held at 25°C and larvae and pupae held at 20°C, as previously described [28].

We created a single normalized cDNA library from all life stages to maximize representation of the *S. crassipalpis *transcriptome. Because we are particularly interested in pupal diapause and reproduction, we included both diapause-destined and direct developing individuals, and among adults we included both protein-fed and protein-starved males and females sampled from eclosion through one reproductive bout (see Table 1 for full sampling scheme). RNA from each sample group was extracted separately in Tri-reagent (Ambion) and the quality of total RNA was verified on 1.4% agarose-MOPS-formaldehyde denaturing gels. Poly-A RNA was extracted from each total RNA sample using the Oligotex^® ^mRNA Midi Kit (Qiagen) and the quality again verified on a denaturing gel. Because the total mRNA yield from each pool differed, 1 μg of mRNA from each sample group was combined into a single large pool and mixed well. This single large, equally-mixed pool was the source of starting material for the creation of the normalized cDNA library. An initial cDNA library was constructed using the SMART cDNA Synthesis Kit (BD Clonetech, Mountain View, CA) and normalized using the Trimmer-Direct Kit (Evrogen, Moscow). The quality of the normalized cDNA library was checked on a 1.4% agarose gel.

Approximately 2.5 μg of the normalized cDNA pool was used for a titration run using one quarter of a plate on the Roche GS FLX sequencer and was followed by a whole plate run on the same equipment using 15 μg of cDNA and a full GS FLX plate at the Interdisciplinary Center for Biotechnology Research at the University of Florida (UF-ICBR). The pyroluminescence intensity for each bead-containing well was used to determine base calls for each nucleotide incorporation step. Sequence assembly was performed first using the Newbler Assembler v1.1.02.15 (454 Life Science, Branford, CT). Afterwards, Newbler output, including all contigs and singletons, was subjected to a second assembly step using the Paracel Transcript Assembler v3.0.0 (Paracel Inc., Pasadena, CA). Sequences were masked for oligonucleotide adaptors used in SMART cDNA synthesis and normalization using a threshold of 15. Low-quality data (base call score < 10) was trimmed from the ends of individual sequences. Low complexity sequence regions (simple sequence repeats) were identified and excluded from consideration during initial pair-wise comparison, but were included during final alignment and consensus building. Assembly was performed in two stages. The first stage used the "Haste" algorithm to build groups (or clusters) of sequences sharing a minimal amount of identifiable sequence similarity (threshold = 50). The second stage assembled sequences within individual clusters into consensus transcripts using the software defaults, except parameters MinCovRep (500), InOverhang (30), EndOverhang (30), RemOverhang (30), QualSumLim (300), MaxInternalGaps (15) and PenalizeN (0). For our dataset, an advantage of the second assembly step using Paracel Transcript Assembler was in cleaning-up the data by further masking any SMART adaptor sequences or poly-A stretches that were included in the initial Newbler assembly.

All contigs and singlets were annotated by BLAST search against NCBI NR and NT databases where the e-value threshold was set at 1e-4. For each query sequence, the top 100 BLAST hits were obtained if available and stored in BlastQuest [29]. If a BLAST hit represented a NCBI Gene database entry, it was used to map *Sarcophaga *transcripts to putative homologs from *Drosophila*, human, mouse, zebrafish, dog or other organisms. Gene Ontology (GO) annotations were also derived based on sequence similarity. Transcripts were classified into 14 major Gene Ontology categories under biological process. If a transcript was annotated with more than one GO category it was split equally among them.

We also used our GO annotations to assess whether coding sequence divergence within Diptera is relatively homogenous across GO categories. We obtained full translation databases for *D. melanogaster *and *A. gambiae *from FlyBase  and VectorBase , respectively. We then used our *S. crassipalpis *EST database as a query in a BlastX search against each of these additional databases setting the e-value threshold to 1 × 10^-4 ^and using default values for all other parameters. The 454 sequencing technology is prone to producing apparent frame-shift errors via erroneous insertions or deletions of extra bases [30], limiting the quality and length of codon alignments and blastX hits. Without correction, codons and proteins will align in overlapping, frame-shifted segments, and to our knowledge no pyrosequencing study has previously addressed this issue. We developed a procedure to identify and correct for 454 insertion (overcall) and deletion (undercall) errors including the following steps: 1) Identify BlastX hits with two frame-shifted, overlapping High-scoring Segment Pairs (HSPs) both with e-values less than 1 × 10^-5 ^(if present, we excluded additional, higher e-value HSPs from our alignments). 2) Determine protein alignment score for each HSP in the region of overlap. We calculated alignment scores by assigning a value of 1 for identical residues, 0.5 for a positive alignment score in the BLOSUM62 scoring matrix, and 0 for non-positive BLOSUM62 scores or a gap. 3) Retain the region of overlap in the HSP alignment with the highest alignment score, and remove the region of overlap from both the un-aligned nucleotide and aligned protein sequence of the lower scoring HSP. 4) Remove one or two additional nucleotides past the region of overlap in the un-aligned nucleotide sequence for the lower-scoring HSP if the frameshift is +1 or +2, respectively. These are nucleotides in the 5' or 3' direction if the lower scoring HSP is more 5' or 3', respectively. 5) Insert a single gap in the query protein sequence at the 3' end of the first HSP (or the 5' end of the second HSP) if the frameshift is +2. 6) Concatenate the highest scoring HSP (protein) with the lowest scoring, truncated HSP. The procedure corrects for single insertion and deletion errors to produce the best scoring full alignment based on our scoring criteria for the region of overlap. In the case of an insertion (+1 frameshift) error, a single nucleotide is removed in the correction. In the case of a deletion (+2 frameshift) two nucleotides are removed and a gap in the protein sequence is introduced. This effectively removes a codon from the alignment, reflecting the uncertainty in reconstructing the translated amino acid associated with the deletion. Out of 12,321 total blastX hits from a *Sarcophaga *to *Drosophila *translation database search, 1036 (8 percent) had putative frame-shift errors, 681 +1 (insertion) shifts and 355 +2 (deletion) shifts. Clearly correcting these frame-shift errors will substantially improve genome-wide alignments using any 454-derived transcriptome.

After applying frame-shift corrections, we retained the top-scoring amino acid alignment from each BlastX hit and produced the associated transcript alignments using tranalign in the EMBOSS package [31] in conjunction with transcription databases for *D. melanogaster *and *A. gambiae*. Using the program YN00 from the PAML package [32] we estimated synonymous (dS) and non-synonymous (dN) substitution rates for each alignment via the method of Yang and Nielsen [33]. Our initial intention was to estimate dN/dS ratios, but alignments were largely oversaturated with synonymous substitution rates (often inestimable). Thus, we retained only dN estimates. Finally, we estimated the median, interquartile range (IQR), and an estimate of the 95% confidence interval of the median as ± 1.58 IQR/sqrt(n) for dN values from each GO category (genes falling into more than one GO category contribute to estimates in all associated categories). This particular 95% CI estimate provides a confidence interval that is largely insensitive to the underlying distribution [34]. We also performed all of the above steps on the pairwise comparison between *D. melanogaster *and *A. gambiae*, yielding three pairwise comparisons in the complete analysis.

## Authors' contributions

DAH conceived the study and design, collected samples, built the libraries, participated in the data analysis and drafted the manuscript. GJR participated in the data analysis and drafted the manuscript. DDS participated in the design of the study, participated in library construction, data analysis, and drafting the manuscript. DLD helped conceive the study, participated in the design and coordination, and drafted the manuscript. All authors read and approved the final manuscript.

## Supplementary Material

Additional file 1 **Examples of potential diapause-related cDNAs identified in this EST library.** Click here for file

Additional file 2 **Microsatellite discovery**. 2a. Summary of potential microsatellite loci identified. 2b. List of contigs and singletons containing potential microsatellite loci including repeat type and length. Click here for file
