# Supplementary material for: Gene discovery using massively parallel pyrosequencing to develop ESTs for the flesh fly Sarcophaga crassipalpis
Source: BMC Genomics. 2009 May 19;10:234. doi: 10.1186/1471-2164-10-234 (PMC2700817; doi:10.1186/1471-2164-10-234)
Supplement: Additional file 1 — Examples of potential diapause-related cDNAs identified in this EST library. [file 1471-2164-10-234-S1.pdf]

Protein Name and Functional Class	Contig no. (E-value <0.0001)
**Endocrine Signaling**	
Juvenile Hormone Esterase	Contigs: 273, 7011, 6101, 1204, 9620, 2797, 4868 8038 16459 16166<br>Singletons: EUA37Q301CA1EB, EUA37Q302GUS7H, EUA37Q301B2UQJ
Juvenile Hormone Epoxide Hydrolase	Contigs: 10882, 994, 8736<br>Singletons: EUA37Q301CBJJU, EUA37Q302HR36D, EUA37Q302HDOVJ
AKH Receptor	Contigs: 6451, 6076,<br>Singletons: EUA37Q301ARTGA, EUA37Q302JPZ9M
Insulin Binding Protein	Contigs: 10659 4515 16613 14165 6683<br>Singleton: EUA37Q301DTKLY
Insulin Receptor	Contig: 7729<br>Singletons: EUA37Q301DLSQH, EUA37Q301C9CKA
Chico (IRS)	Contigs: 13961, 2673,
AKT/PKB	Contigs: 2448, 3001<br>Singleton: EUA37Q302GEQ7G
PTEN	Contigs: 10586, 1699, 10586<br>Singleton: EUA37Q301CUHAB
TOR	Singleton: EUA37Q301EHFWK
TSC1	Singleton: EUA37Q301B88D3
TSC2	Singletons: EUA37Q301BYBIR, EUA37Q301EUOTJ
S6K	Contigs: 5556, 8128<br>Singletons: EUA37Q301CUHAB, EUA37Q302FQNRF, EUA37Q301E2QVJ
FOXO	Contig: 14766

Energy Metabolism

Hexamerin Arylphorin/LSP-1	Contigs: 243 , 3923, 7727<br>Singletons: EUA37Q301DFDSI EUA37Q301DLOU EUA37Q301EM5MZ EUA37Q302FKX17 EUA37Q302GAE1C
Hexamerin LSP-2	Contigs: 10626 14217<br>Singletons: EUA37Q302G46I8 EUA37Q301BDJ92
Arylphorin Receptor	Contigs: 7727, 7765,<br>Singletons: EUA37Q302ISAY8 EUA37Q301B7G20 EUA37Q301B0WZK EUA37Q302GWQFY EUA37Q301EYAAI
Glycogen Phosphorylase	Contigs: 9753, 2930, 7750 10710<br>Singletons: EUA37Q301DYIEG EUA37Q301CA7F1
Glycogen Synthase	Contig: 10189<br>Singleton: EUA37Q301BE2JV
Glycogenenin	Contigs: 9880 9879<br>Singletons: EUA37Q301EKH2J EUA37Q302F4CE6
AMPK-α	Contigs: 4779 2886<br>Singleton: EUA37Q301A1Z02
AMPK-β	Contigs: 9472, 7752,
AMPK-γ	Contig: 3785<br>Singletons: EUA37Q302HPNUL EUA37Q301AS10O EUA37Q301AVUOY
Acetyl-CoA Carboxylase	Contigs: 2701 8433 10857
Fatty Acid Synthase	Contigs: 78 16821 13146 13354<br>Singletons: EUA37Q301AXQK1 EUA37Q302HOIOQ EUA37Q301AYNNB

	EUA37Q301EQHRJ
TAG lipase	Contigs: 10293 16066 16831 12817 12662 7520<br>Singleton: EUA37Q301DMT3Z
Hexokinases	Contigs: 1014 493 11245 1544 8748 12208 16228 12208 16807 15602<br>Singletons: EUA37Q301B5V0H EUA37Q302I4VOH
Phosphofructokinases	Contigs: 9198 2626 3036 16884<br>Singletons: EUA37Q301DJV92 EUA37Q301DFJOJ
Malic Ezyme	Contigs: 3394 3926 317 11562 3902<br>Singletons: EUA37Q301ANUEU
Aspartate Aminotransferase	Contigs: 10486 8960 9481<br>Singletons: EUA37Q301A3ZQN
PEP Carboxylase	Contigs: 1138 9464<br>Singletons: EUA37Q301CJHUS EUA37Q302FY2W5 EUA37Q302G9LSW EUA37Q301CYK72
**Stress Responses/Immunity**	
Catalase	Contigs: 13937, 447,<br>Singleton: EUA37Q301ELU08
Mn Superoxide dismutase	Contig: 10896<br>Singleton: EUA37Q301DIOLX
CuZn Superoxide Dismutase	Contigs: 2923 8932 4020<br>Singletons: EUA37Q301B3WW0 EUA37Q302G08YJ
HSP 40/DNA J	Contigs: 361 606 4072 8860 3909 8680 4506<br>Singletons: EUA37Q301CUQUN EUA37Q302IBWZM
Caspases	Contigs: 4065 8621 1253 2705 15957<br>Singletons: 13446EUA37Q302JC5EF EUA37Q301CBU6O EUA37Q302IG9HA EUA37Q301BUUCL

	EUA37Q301CKSRL
Cactus/I-kappa B	Contigs: 9477 5480
Dorsal/ NF-kappaB	Contigs: 14985 14919 2851<br>Singletons: EUA37Q302I921J<br>EUA37Q302J5X4W
Relish/NF-kappaB-like	Contigs: 5403, 2786, 759, 10400, 2119,<br>10594, 14919,<br>Singleton: EUA37Q302GWQ8U
Defensins	Contigs: 3470 10281 1018<br>Singleton: EUA37Q302GZNOQ
Aquaporins	Contigs: 3534, 2663, 214, 2468, 3453,<br>3534, 15501, 16768, 13680<br>Singletons: EUA37Q302GM7M1<br>EUA37Q302JNYZU EUA37Q301D2TV5<br>EUA37Q301AHAPI EUA37Q301CAJYH<br>EUA37Q301EKYL1<br>EUA37Q301B3QSO EUA37Q302I6YJB<br>EUA37Q301AS1SW
