# Supplementary material for: Gene discovery using massively parallel pyrosequencing to develop ESTs for the flesh fly Sarcophaga crassipalpis
Source: BMC Genomics. 2009 May 19;10:234. doi: 10.1186/1471-2164-10-234 (PMC2700817; doi:10.1186/1471-2164-10-234)
Supplement: Additional file 2 — Microsatellite discovery. 2a. Summary of potential microsatellite loci identified. 2b. List of contigs and singletons containing potential microsatellite loci including repeat type and length. [file 1471-2164-10-234-S2.pdf]

2a. Repeat type	# of hits (repeat length ≥ 7)
dinucleotide	295
trinucleotide	197
tetranucleotide	26
pentanucleotide	1
hexanucleotide	2
total:	521

2b. Sequence ID	Motif type	Repeat
contig01834	Dinucleotide	(AT)^8
contig05641	Dinucleotide	(AC)^7
contig16973	Dinucleotide	(AG)^8
EOHUN8205C5A7Y	Dinucleotide	(AC)^8
EOHUN8205CV4AS	Dinucleotide	(AG)^7
EOHUN8206DKAGE	Dinucleotide	(AT)^7
EOHUN8206DMXVN	Dinucleotide	(AT)^7
EOHUN8206DOIGS	Dinucleotide	(CT)^7
EOHUN8206DRB8M	Dinucleotide	(AG)^7
EUA37Q301A0E6H	Dinucleotide	(GT)^8
EUA37Q301A1F2B	Dinucleotide	(AC)^7
EUA37Q301A7IH8	Dinucleotide	(CT)^8
EUA37Q301A922B	Dinucleotide	(AG)^7
EUA37Q301AE7OS	Dinucleotide	(AT)^7
EUA37Q301AG9XX	Dinucleotide	(AT)^8
EUA37Q301AH1UE	Dinucleotide	(AG)^7
EUA37Q301AHPZ4	Dinucleotide	(CT)^8
EUA37Q301AITNB	Dinucleotide	(CT)^7
EUA37Q301ANFOV	Dinucleotide	(GT)^9
EUA37Q301APRZ9	Dinucleotide	(GT)^8
EUA37Q301AQ9CB	Dinucleotide	(AT)^7
EUA37Q301B0VT3	Dinucleotide	(GT)^8
EUA37Q301B328B	Dinucleotide	(AG)^9
EUA37Q301B5T21	Dinucleotide	(AT)^7
EUA37Q301B74I3	Dinucleotide	(AT)^8
EUA37Q301B74WU	Dinucleotide	(AG)^8
EUA37Q301B7CAV	Dinucleotide	(AT)^8
EUA37Q301B7MQW	Dinucleotide	(CT)^9
EUA37Q301B86XG	Dinucleotide	(AT)^8
EUA37Q301B9O0K	Dinucleotide	(AT)^7
EUA37Q301BAIF3	Dinucleotide	(CT)^9
EUA37Q301BB0FP	Dinucleotide	(GT)^7
EUA37Q301BC61F	Dinucleotide	(CT)^8
EUA37Q301BETVD	Dinucleotide	(AT)^7
EUA37Q301BHM22	Dinucleotide	(AC)^9
EUA37Q301BHR0D	Dinucleotide	(CT)^7
EUA37Q301BJN0I	Dinucleotide	(AT)^8

EUA37Q301BJOIW	Dinucleotide	(AG)^7
EUA37Q301BNJC6	Dinucleotide	(CT)^7
EUA37Q301BVGOT	Dinucleotide	(AG)^7
EUA37Q301BXCMJ	Dinucleotide	(AC)^10
EUA37Q301BYST0	Dinucleotide	(GT)^7
EUA37Q301BZCMD	Dinucleotide	(CT)^8
EUA37Q301C0P0F	Dinucleotide	(CT)^7
EUA37Q301C2VJP	Dinucleotide	(AC)^7
EUA37Q301C84YS	Dinucleotide	(AG)^9
EUA37Q301C9I18	Dinucleotide	(AC)^7
EUA37Q301CATSU	Dinucleotide	(AG)^8
EUA37Q301CCHLJ	Dinucleotide	(CT)^8
EUA37Q301CGOFL	Dinucleotide	(GT)^9
EUA37Q301CHKEL	Dinucleotide	(CT)^8
EUA37Q301CISIG	Dinucleotide	(CT)^7
EUA37Q301CISIG	Dinucleotide	(GT)^8
EUA37Q301CJNRD	Dinucleotide	(GT)^7
EUA37Q301COLYD	Dinucleotide	(AT)^7
EUA37Q301CRH2I	Dinucleotide	(AT)^7
EUA37Q301CRS68	Dinucleotide	(CT)^7
EUA37Q301CU8EH	Dinucleotide	(AC)^12
EUA37Q301CUALA	Dinucleotide	(CT)^9
EUA37Q301CVZJP	Dinucleotide	(AG)^9
EUA37Q301D2OEU	Dinucleotide	(AT)^8
EUA37Q301D3KFI	Dinucleotide	(CT)^7
EUA37Q301D519O	Dinucleotide	(CT)^12
EUA37Q301D6NNO	Dinucleotide	(AC)^7
EUA37Q301DA9AI	Dinucleotide	(AG)^7
EUA37Q301DCNXO	Dinucleotide	(CT)^7
EUA37Q301DFRSO	Dinucleotide	(GT)^10
EUA37Q301DG0XR	Dinucleotide	(AG)^9
EUA37Q301DGES7	Dinucleotide	(AG)^8
EUA37Q301DH8IZ	Dinucleotide	(GT)^7
EUA37Q301DJISF	Dinucleotide	(CT)^7
EUA37Q301DM42W	Dinucleotide	(AT)^7
EUA37Q301DNCRE	Dinucleotide	(GT)^8
EUA37Q301DQYLI	Dinucleotide	(GT)^7
EUA37Q301DS17L	Dinucleotide	(AT)^9
EUA37Q301DU0DN	Dinucleotide	(GT)^7
EUA37Q301DUXW4	Dinucleotide	(AG)^8
EUA37Q301DVMTL	Dinucleotide	(AC)^11
EUA37Q301DX35X	Dinucleotide	(AC)^7
EUA37Q301DZUBH	Dinucleotide	(AG)^7
EUA37Q301E0JDD	Dinucleotide	(AT)^7
EUA37Q301E2NZI	Dinucleotide	(GT)^9
EUA37Q301EALU5	Dinucleotide	(AG)^9

EUA37Q301EALU5	Dinucleotide	(AG)^9
EUA37Q301EF27U	Dinucleotide	(AC)^8
EUA37Q301EHD57	Dinucleotide	(AC)^8
EUA37Q301EIC8Y	Dinucleotide	(AT)^8
EUA37Q301ENI7R	Dinucleotide	(AT)^7
EUA37Q301EPLTE	Dinucleotide	(AT)^7
EUA37Q301ER611	Dinucleotide	(AG)^8
EUA37Q301ESDRF	Dinucleotide	(AT)^8
EUA37Q301EV8JS	Dinucleotide	(AC)^9
EUA37Q301EXCZ2	Dinucleotide	(CT)^7
EUA37Q302F0CHI	Dinucleotide	(AT)^7
EUA37Q302F1UVM	Dinucleotide	(AC)^7
EUA37Q302F2MV6	Dinucleotide	(AG)^15
EUA37Q302FH4UT	Dinucleotide	(CT)^8
EUA37Q302FM3RP	Dinucleotide	(AT)^7
EUA37Q302FMY5E	Dinucleotide	(GT)^10
EUA37Q302FOZPB	Dinucleotide	(AT)^8
EUA37Q302FZJV2	Dinucleotide	(AC)^7
EUA37Q302G1RTN	Dinucleotide	(AC)^9
EUA37Q302G3RN7	Dinucleotide	(AC)^7
EUA37Q302G3UX5	Dinucleotide	(CT)^8
EUA37Q302G44N6	Dinucleotide	(AG)^9
EUA37Q302G7F9B	Dinucleotide	(AC)^7
EUA37Q302G93N6	Dinucleotide	(AT)^8
EUA37Q302GDHWR	Dinucleotide	(CT)^7
EUA37Q302GFNTM	Dinucleotide	(AC)^7
EUA37Q302GGRMN	Dinucleotide	(GT)^9
EUA37Q302GIUP9	Dinucleotide	(AG)^7
EUA37Q302GOD8U	Dinucleotide	(AT)^7
EUA37Q302GPC7A	Dinucleotide	(GT)^12
EUA37Q302GT4WN	Dinucleotide	(AT)^7
EUA37Q302GTRX6	Dinucleotide	(CT)^17
EUA37Q302H1C9Q	Dinucleotide	(AC)^7
EUA37Q302H1NH2	Dinucleotide	(AT)^7
EUA37Q302H2M8N	Dinucleotide	(CT)^9
EUA37Q302H36GZ	Dinucleotide	(AT)^7
EUA37Q302H4K3I	Dinucleotide	(AG)^7
EUA37Q302H75JZ	Dinucleotide	(AC)^7
EUA37Q302H9JCB	Dinucleotide	(CT)^7
EUA37Q302HCMGG	Dinucleotide	(GT)^7
EUA37Q302HEY6I	Dinucleotide	(AT)^7
EUA37Q302HHJGR	Dinucleotide	(CT)^10
EUA37Q302HI1OH	Dinucleotide	(AG)^10
EUA37Q302HOKNH	Dinucleotide	(CT)^8
EUA37Q302HSAT7	Dinucleotide	(CT)^8
EUA37Q302HSP86	Dinucleotide	(CT)^9

EUA37Q302HTESY	Dinucleotide	(AC)^10
EUA37Q302HTJTP	Dinucleotide	(CT)^11
EUA37Q302HU9PF	Dinucleotide	(AC)^7
EUA37Q302HUXL4	Dinucleotide	(AG)^8
EUA37Q302HWJHC	Dinucleotide	(AC)^7
EUA37Q302I05YZ	Dinucleotide	(CT)^13
EUA37Q302I0JVD	Dinucleotide	(GT)^7
EUA37Q302I2Y9Z	Dinucleotide	(AG)^21
EUA37Q302I5DLQ	Dinucleotide	(GT)^8
EUA37Q302I6A2Q	Dinucleotide	(AG)^8
EUA37Q302I7UUD	Dinucleotide	(AG)^10
EUA37Q302I85DB	Dinucleotide	(GT)^15
EUA37Q302IAMW1	Dinucleotide	(GT)^10
EUA37Q302IBZEA	Dinucleotide	(GT)^9
EUA37Q302IGJ09	Dinucleotide	(GT)^11
EUA37Q302IGW8Y	Dinucleotide	(AC)^12
EUA37Q302IJA1I	Dinucleotide	(AC)^7
EUA37Q302ILSOZ	Dinucleotide	(AT)^7
EUA37Q302ILY0U	Dinucleotide	(AT)^8
EUA37Q302IPSMY	Dinucleotide	(CT)^9
EUA37Q302IQZQP	Dinucleotide	(AC)^7
EUA37Q302IRDRT	Dinucleotide	(CT)^9
EUA37Q302IRHWJ	Dinucleotide	(AT)^9
EUA37Q302ITNZL	Dinucleotide	(AC)^7
EUA37Q302IVJSC	Dinucleotide	(AG)^7
EUA37Q302IVTBY	Dinucleotide	(CT)^11
EUA37Q302IW538	Dinucleotide	(AT)^9
EUA37Q302IXFYU	Dinucleotide	(GT)^10
EUA37Q302IYXTS	Dinucleotide	(CT)^9
EUA37Q302IZAL7	Dinucleotide	(AT)^8
EUA37Q302J384I	Dinucleotide	(AC)^7
EUA37Q302JCKDU	Dinucleotide	(AC)^9
EUA37Q302JFU3X	Dinucleotide	(AT)^7
EUA37Q302JFWAM	Dinucleotide	(AC)^8
EUA37Q302JGIYY	Dinucleotide	(CT)^10
EUA37Q302JIEVP	Dinucleotide	(CT)^7
EUA37Q302JKBA3	Dinucleotide	(CT)^7
EUA37Q302JOPQY	Dinucleotide	(GT)^8
EUA37Q302JTKEA	Dinucleotide	(AC)^7
EUA37Q302JWSTN	Dinucleotide	(CT)^11
HAHN.FLY.10068.C1	Dinucleotide	(AC)^8
HAHN.FLY.10134.C1	Dinucleotide	(AT)^7
HAHN.FLY.10138.C2	Dinucleotide	(AT)^10
HAHN.FLY.10210.C1	Dinucleotide	(AG)^7
HAHN.FLY.10210.C5	Dinucleotide	(AG)^7
HAHN.FLY.10284.C1	Dinucleotide	(GT)^7

HAHN.FLY.10304.C1	Dinucleotide	(AG)^8
HAHN.FLY.10396.C1	Dinucleotide	(AG)^7
HAHN.FLY.1079.C1	Dinucleotide	(AC)^7
HAHN.FLY.10850.C1	Dinucleotide	(AT)^8
HAHN.FLY.10850.C3	Dinucleotide	(AT)^7
HAHN.FLY.10858.C5	Dinucleotide	(AT)^8
HAHN.FLY.10945.C1	Dinucleotide	(AG)^7
HAHN.FLY.11146.C1	Dinucleotide	(CT)^7
HAHN.FLY.11210.C1	Dinucleotide	(GT)^7
HAHN.FLY.11361.C1	Dinucleotide	(CT)^10
HAHN.FLY.11521.C1	Dinucleotide	(CT)^9
HAHN.FLY.11568.C1	Dinucleotide	(GT)^7
HAHN.FLY.1164.C1	Dinucleotide	(CT)^10
HAHN.FLY.11901.C1	Dinucleotide	(GT)^8
HAHN.FLY.11987.C1	Dinucleotide	(CT)^7
HAHN.FLY.12103.C1	Dinucleotide	(AT)^9
HAHN.FLY.12260.C1	Dinucleotide	(AC)^7
HAHN.FLY.12263.C1	Dinucleotide	(CT)^7
HAHN.FLY.12429.C1	Dinucleotide	(GT)^7
HAHN.FLY.12511.C1	Dinucleotide	(AT)^7
HAHN.FLY.12704.C1	Dinucleotide	(AC)^7
HAHN.FLY.12732.C1	Dinucleotide	(AC)^7
HAHN.FLY.13171.C1	Dinucleotide	(AG)^7
HAHN.FLY.13268.C1	Dinucleotide	(AC)^8
HAHN.FLY.13428.C1	Dinucleotide	(AG)^7
HAHN.FLY.13490.C1	Dinucleotide	(GT)^9
HAHN.FLY.13686.C1	Dinucleotide	(AT)^7
HAHN.FLY.1378.C1	Dinucleotide	(GT)^7
HAHN.FLY.1378.C2	Dinucleotide	(AC)^9
HAHN.FLY.1378.C2	Dinucleotide	(AC)^9
HAHN.FLY.14031.C1	Dinucleotide	(AT)^8
HAHN.FLY.14220.C1	Dinucleotide	(AC)^8
HAHN.FLY.14346.C1	Dinucleotide	(AT)^7
HAHN.FLY.14357.C1	Dinucleotide	(CT)^8
HAHN.FLY.14722.C1	Dinucleotide	(CT)^7
HAHN.FLY.14797.C1	Dinucleotide	(AT)^7
HAHN.FLY.14973.C1	Dinucleotide	(AT)^7
HAHN.FLY.15.C3	Dinucleotide	(AT)^7
HAHN.FLY.15488.C1	Dinucleotide	(AG)^8
HAHN.FLY.15837.C1	Dinucleotide	(AT)^9
HAHN.FLY.15896.C1	Dinucleotide	(CT)^7
HAHN.FLY.16366.C1	Dinucleotide	(AT)^7
HAHN.FLY.1654.C1	Dinucleotide	(AC)^7
HAHN.FLY.16896.C1	Dinucleotide	(AG)^8
HAHN.FLY.1715.C1	Dinucleotide	(AC)^7
HAHN.FLY.172.C4	Dinucleotide	(CT)^8

HAHN.FLY.17282.C1	Dinucleotide	(AT)^8
HAHN.FLY.17433.C1	Dinucleotide	(AC)^10
HAHN.FLY.17469.C1	Dinucleotide	(AG)^7
HAHN.FLY.1939.C4	Dinucleotide	(CT)^9
HAHN.FLY.2001.C1	Dinucleotide	(CT)^8
HAHN.FLY.2051.C1	Dinucleotide	(CT)^12
HAHN.FLY.2084.C1	Dinucleotide	(AT)^7
HAHN.FLY.2231.C1	Dinucleotide	(AC)^10
HAHN.FLY.2304.C1	Dinucleotide	(AG)^8
HAHN.FLY.2479.C1	Dinucleotide	(AC)^8
HAHN.FLY.2507.C1	Dinucleotide	(CT)^9
HAHN.FLY.2727.C1	Dinucleotide	(GT)^7
HAHN.FLY.2896.C1	Dinucleotide	(AG)^7
HAHN.FLY.3056.C1	Dinucleotide	(GT)^9
HAHN.FLY.3107.C1	Dinucleotide	(GT)^7
HAHN.FLY.3109.C1	Dinucleotide	(AG)^10
HAHN.FLY.3196.C2	Dinucleotide	(AC)^7
HAHN.FLY.3480.C1	Dinucleotide	(AG)^7
HAHN.FLY.3815.C1	Dinucleotide	(CT)^7
HAHN.FLY.3861.C1	Dinucleotide	(AC)^7
HAHN.FLY.3893.C1	Dinucleotide	(AC)^7
HAHN.FLY.4313.C1	Dinucleotide	(AC)^7
HAHN.FLY.461.C1	Dinucleotide	(AT)^7
HAHN.FLY.4672.C1	Dinucleotide	(CT)^7
HAHN.FLY.4777.C1	Dinucleotide	(AG)^7
HAHN.FLY.485.C1	Dinucleotide	(AC)^7
HAHN.FLY.4863.C1	Dinucleotide	(GT)^11
HAHN.FLY.492.C1	Dinucleotide	(CT)^10
HAHN.FLY.4933.C1	Dinucleotide	(AG)^7
HAHN.FLY.5.C4	Dinucleotide	(GT)^8
HAHN.FLY.5.C5	Dinucleotide	(AC)^8
HAHN.FLY.5027.C1	Dinucleotide	(AC)^8
HAHN.FLY.5400.C1	Dinucleotide	(AG)^7
HAHN.FLY.5867.C1	Dinucleotide	(AC)^7
HAHN.FLY.5909.C1	Dinucleotide	(AC)^10
HAHN.FLY.6025.C1	Dinucleotide	(AT)^7
HAHN.FLY.6156.C1	Dinucleotide	(AG)^8
HAHN.FLY.6164.C1	Dinucleotide	(AC)^7
HAHN.FLY.6176.C1	Dinucleotide	(AT)^7
HAHN.FLY.6203.C1	Dinucleotide	(GT)^8
HAHN.FLY.6383.C1	Dinucleotide	(GT)^7
HAHN.FLY.6411.C1	Dinucleotide	(AC)^7
HAHN.FLY.6421.C1	Dinucleotide	(CT)^8
HAHN.FLY.6463.C1	Dinucleotide	(CT)^7
HAHN.FLY.6475.C1	Dinucleotide	(AC)^8
HAHN.FLY.6803.C2	Dinucleotide	(AG)^8

HAHN.FLY.6813.C1	Dinucleotide	(AG)^7
HAHN.FLY.6873.C1	Dinucleotide	(AT)^8
HAHN.FLY.7197.C1	Dinucleotide	(AT)^9
HAHN.FLY.7578.C1	Dinucleotide	(GT)^8
HAHN.FLY.7614.C1	Dinucleotide	(AC)^9
HAHN.FLY.7722.C1	Dinucleotide	(AT)^10
HAHN.FLY.7891.C2	Dinucleotide	(AG)^7
HAHN.FLY.7896.C1	Dinucleotide	(AC)^7
HAHN.FLY.7963.C1	Dinucleotide	(GT)^8
HAHN.FLY.7972.C1	Dinucleotide	(CT)^12
HAHN.FLY.8556.C1	Dinucleotide	(AT)^8
HAHN.FLY.8649.C1	Dinucleotide	(AG)^8
HAHN.FLY.8695.C1	Dinucleotide	(AC)^7
HAHN.FLY.8746.C1	Dinucleotide	(AG)^9
HAHN.FLY.877.C1	Dinucleotide	(AT)^7
HAHN.FLY.8927.C4	Dinucleotide	(AG)^8
HAHN.FLY.9108.C1	Dinucleotide	(AC)^9
HAHN.FLY.9203.C2	Dinucleotide	(AG)^7
HAHN.FLY.9300.C1	Dinucleotide	(CT)^9
HAHN.FLY.9358.C1	Dinucleotide	(CT)^7
HAHN.FLY.9460.C1	Dinucleotide	(GT)^9
HAHN.FLY.9496.C1	Dinucleotide	(AC)^7
HAHN.FLY.9530.C6	Dinucleotide	(GT)^8
HAHN.FLY.9539.C2	Dinucleotide	(AT)^7
HAHN.FLY.9585.C1	Dinucleotide	(AC)^10
HAHN.FLY.9842.C4	Dinucleotide	(GT)^9
HAHN.FLY.9920.C1	Dinucleotide	(AG)^9
HAHN.FLY.9973.C2	Dinucleotide	(AT)^9
EOHUN8206DIXU6	Trinucleotide	(GAT)^9
EOHUN8206DKXZL	Trinucleotide	(CTT)^7
EOHUN8206DRS1P	Trinucleotide	(AAT)^7
EUA37Q301A60JL	Trinucleotide	(AAT)^7
EUA37Q301AQOZK	Trinucleotide	(GAT)^7
EUA37Q301ARDRU	Trinucleotide	(AGT)^7
EUA37Q301ARDRU	Trinucleotide	(GGT)^7
EUA37Q301AV5V0	Trinucleotide	(ATT)^7
EUA37Q301B2MOC	Trinucleotide	(AGT)^8
EUA37Q301B2ORA	Trinucleotide	(GTT)^9
EUA37Q301B4B56	Trinucleotide	(GTT)^7
EUA37Q301B4BFR	Trinucleotide	(GAT)^7
EUA37Q301BB53N	Trinucleotide	(AAC)^9
EUA37Q301BBFJU	Trinucleotide	(GTT)^7
EUA37Q301BGLSF	Trinucleotide	(AAC)^7
EUA37Q301BJH6U	Trinucleotide	(ATT)^7
EUA37Q301BKQBP	Trinucleotide	(ACC)^7

EUA37Q301BLBAO	Trinucleotide	(GTT)^8
EUA37Q301BN1U6	Trinucleotide	(CTT)^7
EUA37Q301BRGPV	Trinucleotide	(AAC)^7
EUA37Q301BW7ZX	Trinucleotide	(ATT)^8
EUA37Q301BZPEU	Trinucleotide	(AAC)^8
EUA37Q301C18JR	Trinucleotide	(AAC)^8
EUA37Q301C7MZG	Trinucleotide	(GTT)^9
EUA37Q301C98M4	Trinucleotide	(AAC)^7
EUA37Q301C9VPS	Trinucleotide	(AAC)^7
EUA37Q301CBPRD	Trinucleotide	(ACC)^9
EUA37Q301CDX2M	Trinucleotide	(AAC)^8
EUA37Q301CEWOP	Trinucleotide	(AAC)^9
EUA37Q301COL1L	Trinucleotide	(AAC)^7
EUA37Q301CRN90	Trinucleotide	(AAC)^8
EUA37Q301D34N6	Trinucleotide	(ACT)^8
EUA37Q301D3ZIK	Trinucleotide	(GTT)^7
EUA37Q301DCL88	Trinucleotide	(AAT)^7
EUA37Q301DDPLO	Trinucleotide	(AAC)^9
EUA37Q301DF01X	Trinucleotide	(GTT)^9
EUA37Q301DG4GQ	Trinucleotide	(GTT)^7
EUA37Q301DGG6N	Trinucleotide	(GTT)^7
EUA37Q301DMMW8	Trinucleotide	(AAC)^7
EUA37Q301DSIK8	Trinucleotide	(AAT)^8
EUA37Q301E52CK	Trinucleotide	(AAT)^7
EUA37Q301EAFV6	Trinucleotide	(ATT)^8
EUA37Q301EEA4R	Trinucleotide	(ACT)^7
EUA37Q301EHHNO	Trinucleotide	(AAC)^7
EUA37Q301EJJ0E	Trinucleotide	(AAC)^8
EUA37Q301EKG8U	Trinucleotide	(AAT)^8
EUA37Q301ETCO8	Trinucleotide	(GAT)^8
EUA37Q302F62XK	Trinucleotide	(ATC)^12
EUA37Q302F6A32	Trinucleotide	(GTT)^8
EUA37Q302F8XLC	Trinucleotide	(AAC)^7
EUA37Q302FN2BU	Trinucleotide	(AAC)^8
EUA37Q302FQW8X	Trinucleotide	(AGT)^8
EUA37Q302FVVUF	Trinucleotide	(AGT)^8
EUA37Q302G52EH	Trinucleotide	(GTT)^7
EUA37Q302GA197	Trinucleotide	(AAC)^7
EUA37Q302GCXR4	Trinucleotide	(AAT)^7
EUA37Q302GEBNS	Trinucleotide	(ACG)^7
EUA37Q302GHUG1	Trinucleotide	(GTT)^7
EUA37Q302GKG4S	Trinucleotide	(GTT)^8
EUA37Q302GMY38	Trinucleotide	(AAC)^8
EUA37Q302GTL0P	Trinucleotide	(GTT)^7
EUA37Q302HD58X	Trinucleotide	(ACT)^7
EUA37Q302HDXIX	Trinucleotide	(GCT)^8

EUA37Q302HFWLQ	Trinucleotide	(AAC)^7
EUA37Q302HGO6Y	Trinucleotide	(GTT)^8
EUA37Q302HGWHJ	Trinucleotide	(GTT)^7
EUA37Q302HH9WU	Trinucleotide	(GGT)^10
EUA37Q302HHFIH	Trinucleotide	(GTT)^7
EUA37Q302HHTPV	Trinucleotide	(AGT)^10
EUA37Q302HLPLU	Trinucleotide	(AAC)^8
EUA37Q302HMOFW	Trinucleotide	(AAG)^9
EUA37Q302HQIW7	Trinucleotide	(AAC)^7
EUA37Q302I0GZD	Trinucleotide	(AAC)^12
EUA37Q302I0I6S	Trinucleotide	(GTT)^7
EUA37Q302I0I6S	Trinucleotide	(GTT)^7
EUA37Q302IDZE2	Trinucleotide	(AAC)^7
EUA37Q302ILD42	Trinucleotide	(GTT)^7
EUA37Q302IQ7PC	Trinucleotide	(AGT)^9
EUA37Q302J04OX	Trinucleotide	(GTT)^9
EUA37Q302J159U	Trinucleotide	(AAC)^7
EUA37Q302J1TBY	Trinucleotide	(AAC)^8
EUA37Q302J5WD0	Trinucleotide	(AAC)^7
EUA37Q302JASX0	Trinucleotide	(ATC)^7
EUA37Q302JFGCQ	Trinucleotide	(ATT)^7
EUA37Q302JNUAB	Trinucleotide	(ACT)^7
EUA37Q302JOFXW	Trinucleotide	(GTT)^7
EUA37Q302JT90O	Trinucleotide	(ATC)^8
EUA37Q302JXD77	Trinucleotide	(GAT)^7
HAHN.FLY.10055.C2	Trinucleotide	(GAT)^7
HAHN.FLY.10149.C2	Trinucleotide	(AAT)^7
HAHN.FLY.10276.C1	Trinucleotide	(AAC)^7
HAHN.FLY.10324.C1	Trinucleotide	(AAC)^7
HAHN.FLY.10399.C1	Trinucleotide	(AAC)^9
HAHN.FLY.10476.C1	Trinucleotide	(ATT)^7
HAHN.FLY.10528.C1	Trinucleotide	(GTT)^8
HAHN.FLY.10528.C2	Trinucleotide	(GTT)^7
HAHN.FLY.10731.C1	Trinucleotide	(AAC)^7
HAHN.FLY.10749.C2	Trinucleotide	(ATT)^7
HAHN.FLY.10829.C2	Trinucleotide	(AAC)^9
HAHN.FLY.10850.C1	Trinucleotide	(AAT)^8
HAHN.FLY.10924.C1	Trinucleotide	(AAT)^7
HAHN.FLY.11064.C1	Trinucleotide	(GTT)^7
HAHN.FLY.11085.C1	Trinucleotide	(AAC)^7
HAHN.FLY.11198.C1	Trinucleotide	(AAC)^9
HAHN.FLY.11436.C1	Trinucleotide	(AAT)^7
HAHN.FLY.1164.C2	Trinucleotide	(AAC)^7
HAHN.FLY.11641.C1	Trinucleotide	(AAC)^7
HAHN.FLY.11694.C1	Trinucleotide	(AAC)^7
HAHN.FLY.12358.C1	Trinucleotide	(AAC)^9

HAHN.FLY.12387.C1	Trinucleotide	(AAC)^7
HAHN.FLY.12415.C1	Trinucleotide	(GTT)^8
HAHN.FLY.12521.C1	Trinucleotide	(AAC)^9
HAHN.FLY.12586.C1	Trinucleotide	(AAC)^7
HAHN.FLY.12912.C1	Trinucleotide	(AAC)^7
HAHN.FLY.1301.C1	Trinucleotide	(AAT)^7
HAHN.FLY.13073.C1	Trinucleotide	(AAC)^8
HAHN.FLY.13191.C1	Trinucleotide	(AAT)^8
HAHN.FLY.13300.C1	Trinucleotide	(AAC)^8
HAHN.FLY.13323.C1	Trinucleotide	(AAC)^7
HAHN.FLY.137.C3	Trinucleotide	(AAC)^7
HAHN.FLY.14083.C1	Trinucleotide	(ACT)^12
HAHN.FLY.14227.C1	Trinucleotide	(ATC)^7
HAHN.FLY.14296.C1	Trinucleotide	(AAC)^12
HAHN.FLY.14306.C1	Trinucleotide	(AAC)^8
HAHN.FLY.1431.C2	Trinucleotide	(AAC)^9
HAHN.FLY.14675.C1	Trinucleotide	(ACC)^8
HAHN.FLY.14719.C1	Trinucleotide	(AAC)^8
HAHN.FLY.15381.C1	Trinucleotide	(AAC)^7
HAHN.FLY.154.C1	Trinucleotide	(AAC)^7
HAHN.FLY.15650.C1	Trinucleotide	(ACT)^8
HAHN.FLY.1593.C5	Trinucleotide	(AAC)^8
HAHN.FLY.16446.C1	Trinucleotide	(AAC)^12
HAHN.FLY.1654.C1	Trinucleotide	(GTT)^7
HAHN.FLY.17160.C1	Trinucleotide	(ACG)^7
HAHN.FLY.1770.C4	Trinucleotide	(AAT)^7
HAHN.FLY.1936.C3	Trinucleotide	(ATT)^7
HAHN.FLY.2159.C1	Trinucleotide	(AAC)^8
HAHN.FLY.2159.C1	Trinucleotide	(AAC)^9
HAHN.FLY.226.C1	Trinucleotide	(AAC)^7
HAHN.FLY.245.C1	Trinucleotide	(ATC)^11
HAHN.FLY.2516.C3	Trinucleotide	(AAC)^8
HAHN.FLY.2613.C1	Trinucleotide	(ACT)^7
HAHN.FLY.2902.C1	Trinucleotide	(AAC)^7
HAHN.FLY.2965.C3	Trinucleotide	(AAC)^7
HAHN.FLY.3538.C1	Trinucleotide	(AGC)^7
HAHN.FLY.358.C1	Trinucleotide	(AAC)^7
HAHN.FLY.37.C17	Trinucleotide	(ACT)^9
HAHN.FLY.37.C4	Trinucleotide	(ACC)^9
HAHN.FLY.3705.C1	Trinucleotide	(GGT)^7
HAHN.FLY.3791.C2	Trinucleotide	(AAC)^9
HAHN.FLY.381.C1	Trinucleotide	(AAC)^8
HAHN.FLY.3823.C1	Trinucleotide	(AAC)^8
HAHN.FLY.3833.C1	Trinucleotide	(GTT)^11
HAHN.FLY.3981.C1	Trinucleotide	(AAC)^7
HAHN.FLY.4072.C1	Trinucleotide	(AAC)^10

HAHN.FLY.4072.C2	Trinucleotide	(GTT)^12
HAHN.FLY.4110.C1	Trinucleotide	(GCT)^8
HAHN.FLY.4127.C2	Trinucleotide	(GTT)^9
HAHN.FLY.4208.C1	Trinucleotide	(AAC)^7
HAHN.FLY.4234.C1	Trinucleotide	(ACT)^8
HAHN.FLY.437.C2	Trinucleotide	(AAC)^7
HAHN.FLY.457.C1	Trinucleotide	(AAC)^8
HAHN.FLY.4590.C1	Trinucleotide	(AAT)^7
HAHN.FLY.4700.C1	Trinucleotide	(AAC)^9
HAHN.FLY.5089.C1	Trinucleotide	(AAC)^7
HAHN.FLY.5257.C1	Trinucleotide	(GTT)^7
HAHN.FLY.5423.C1	Trinucleotide	(AAC)^9
HAHN.FLY.547.C1	Trinucleotide	(AAC)^9
HAHN.FLY.5528.C1	Trinucleotide	(AAC)^7
HAHN.FLY.558.C2	Trinucleotide	(AAC)^8
HAHN.FLY.57.C2	Trinucleotide	(GTT)^8
HAHN.FLY.5954.C1	Trinucleotide	(AAC)^7
HAHN.FLY.606.C4	Trinucleotide	(AAT)^7
HAHN.FLY.6086.C1	Trinucleotide	(ATT)^9
HAHN.FLY.6203.C1	Trinucleotide	(AAT)^7
HAHN.FLY.6292.C2	Trinucleotide	(GAT)^7
HAHN.FLY.685.C1	Trinucleotide	(AAC)^11
HAHN.FLY.7061.C1	Trinucleotide	(AAC)^7
HAHN.FLY.7167.C1	Trinucleotide	(AAT)^7
HAHN.FLY.7494.C1	Trinucleotide	(ACC)^7
HAHN.FLY.7727.C3	Trinucleotide	(GAT)^7
HAHN.FLY.775.C1	Trinucleotide	(AAC)^7
HAHN.FLY.775.C1	Trinucleotide	(AAC)^7
HAHN.FLY.7969.C1	Trinucleotide	(GTT)^7
HAHN.FLY.8121.C1	Trinucleotide	(GTT)^9
HAHN.FLY.8706.C1	Trinucleotide	(AAC)^7
HAHN.FLY.8721.C1	Trinucleotide	(AAC)^8
HAHN.FLY.8811.C1	Trinucleotide	(AAT)^7
HAHN.FLY.8924.C3	Trinucleotide	(AAC)^7
HAHN.FLY.9005.C1	Trinucleotide	(AAC)^7
HAHN.FLY.9028.C3	Trinucleotide	(AAC)^7
HAHN.FLY.913.C1	Trinucleotide	(AAC)^7
HAHN.FLY.9298.C1	Trinucleotide	(AAC)^7
HAHN.FLY.9298.C2	Trinucleotide	(AAC)^7
HAHN.FLY.9461.C1	Trinucleotide	(CGT)^7
HAHN.FLY.9461.C1	Trinucleotide	(ATC)^7
HAHN.FLY.9865.C2	Trinucleotide	(AAT)^8
EUA37Q301AW8LX	Tetranucleotide	(ATGT)^7
EUA37Q301BA7UW	Tetranucleotide	(ACAT)^9
EUA37Q301BDRWD	Tetranucleotide	(AAAT)^8

EUA37Q301BK581	Tetranucleotide	(ACAT)^34
EUA37Q301BXXA7	Tetranucleotide	(ATGT)^7
EUA37Q301C8MLP	Tetranucleotide	(ATGT)^7
EUA37Q301CCKD6	Tetranucleotide	(ATGT)^7
EUA37Q301DUG7D	Tetranucleotide	(ATGT)^33
EUA37Q301E6UTO	Tetranucleotide	(ATGT)^7
EUA37Q301EL3SU	Tetranucleotide	(ATGT)^7
EUA37Q302F1I65	Tetranucleotide	(ATGT)^7
EUA37Q302HAPJO	Tetranucleotide	(CTTT)^7
EUA37Q302HTO21	Tetranucleotide	(ACAT)^8
EUA37Q302HV3AM	Tetranucleotide	(CAGT)^7
EUA37Q302I90KE	Tetranucleotide	(ATGT)^7
EUA37Q302ILY06	Tetranucleotide	(ATGT)^7
EUA37Q302IQ0LY	Tetranucleotide	(ATGT)^7
HAHN.FLY.11854.C1	Tetranucleotide	(ATGT)^7
HAHN.FLY.13120.C1	Tetranucleotide	(ATGT)^7
HAHN.FLY.13182.C1	Tetranucleotide	(ATGT)^7
HAHN.FLY.13401.C1	Tetranucleotide	(ACAT)^7
HAHN.FLY.16311.C1	Tetranucleotide	(ATGT)^10
HAHN.FLY.432.C1	Tetranucleotide	(ACAT)^8
HAHN.FLY.5823.C1	Tetranucleotide	(ACAT)^8
HAHN.FLY.7568.C1	Tetranucleotide	(ACAT)^7
HAHN.FLY.8348.C1	Tetranucleotide	(AAAT)^7
HAHN.FLY.9066.C1	Pentanucleotide	(AACGT)^9
HAHN.FLY.162.C2	Hexanucleotide	(AATGAC)^8
HAHN.FLY.714.C1	Hexanucleotide	(AAAATG)^11
